# Participant Experiences with Human Biomonitoring in Communities Affected by Chronic PFAS Environmental Contamination in the Veneto Region (Italy)

**DOI:** 10.3390/ijerph22081190

**Published:** 2025-07-29

**Authors:** Marialuisa Menegatto, Andrea Bobbio, Gloria Freschi, Francesca Celeste Conti, Maria Cristina Cola, Michela Zamboni, Adriano Zamperini

**Affiliations:** 1 FISPPA Department, University of Padova, Via Venezia 14, 35131 Padova, Italy; andrea.bobbio@unipd.it (A.B.); gloria.freschi@phd.unipd.it (G.F.); francescacelesteconti@gmail.com (F.C.C.); 2 Interuniversity Research Centre in Environmental Psychology (CIRPA), 00185 Rome, Italy; 3 Mamme NO PFAS Vicenza, Padova and Verona, 36045 Italy; cristina.cola@mammenopfas.org (M.C.C.); michela.zamboni@mammenopfas.org (M.Z.)

**Keywords:** risk communication, participatory research, environmental health, public health, social psychology

## Abstract

This exploratory study investigated how health concerns related to chronic environmental contamination and how satisfaction with the human biomonitoring (HBM) process influence the perceived quality of life in the context of per- and polyfluoroalkyl substances (PFAS) contamination in the Veneto Region (Italy). We administered a questionnaire to 84 residents of the Red Area, where PFAS exposure is classified as most severe. The main findings revealed that satisfaction with HBM was positively correlated with perceived quality of life and showed a statistically significant but modest moderation effect on the relationship between PFAS-related health concerns and quality of life (explaining 17.4% of the variance). Particularly, it attenuates the negative effect that PFAS health concerns have on quality of life. Differences between subgroups revealed heightened concern regarding PFAS health risks among women vs. men and participants with children vs. those without. These results underscore the central role of relational and communication aspects of HBM programs to mitigate psychological distress and possibly contribute to higher perceived well-being. The study highlights the need for tailored public health interventions, including transparent communication, empathetic support, and community engagement, to address the psychosocial dimensions of environmental contamination.

## 1. Introduction

Human biomonitoring (HBM), which involves measuring chemical substances in the human body, is emerging as a crucial tool for assessing environmental exposure and informing prevention strategies [1]. This scientific approach enables the direct identification of environmental contaminants in biological fluids, such as blood, urine, breast milk, and hair, providing a more accurate measurement compared to traditional environmental assessments based on air, water, or soil analysis [2]. Some of the most frequently monitored substances include heavy metals such as lead and mercury, organochlorine pesticides, per- and polyfluoroalkyl substances (PFAS), and phthalates, which are known for their toxic and potentially endocrine-disrupting effects [3,4,5].

One of the most significant aspects of HBM is its ability to highlight differences in exposure between population groups, enabling the identification of the most vulnerable categories, such as children [6], pregnant women [7], and workers exposed to specific chemicals [8]. For instance, epidemiological studies have shown that prenatal exposure to certain pollutants, as measured through biomonitoring, can negatively affect children’s neurological development [9], underscoring the need for stricter regulatory interventions. Existing regulatory frameworks may not fully capture the complexity and long-term risks associated with widespread and persistent contaminants such as PFAS, leaving significant gaps in protection, especially for vulnerable populations [10,11].

Beyond its usefulness in scientific research, HBM has become a cornerstone in shaping public health policies. The data collected are used to establish reference values for the general population, assess the effectiveness of environmental regulations, and identify trends over time [12]. A significant example is the use of HBM to track the decline in blood lead levels in the U.S. population following the removal of lead from gasoline, demonstrating the positive impact of environmental policies [13].

In today’s world, rapid anthropogenic changes to both natural and built environments, as well as the rapid acceleration in the introduction of new compounds, have significantly increased human exposure to a wide range of chemicals, posing substantial health risks. This exposure is not only extensive but also chronic and complex, as highlighted by recent assessments from international health authorities [14,15], and involves a diverse array of biologically active substances with exposure routes ranging from direct consumer product use to bioaccumulation in the food chain and contamination of groundwater—resulting in long-term exposures that are challenging to characterize fully [16,17]. Managing these risks requires a comprehensive approach that accounts for combined exposures from multiple sources, including both individual chemicals and their mixtures, as well as other environmental stressors.

Recognizing this challenge, the Human Biomonitoring for Europe (HBM4EU) project in Europe has been a key initiative to harmonize data across countries, providing a scientific basis for policy decisions aimed at reducing exposure to hazardous substances [18]. This illustrates how HBM is not just a measurement tool but also a powerful means of prevention and public health protection. With the continuous emergence of new synthetic chemicals, integrating biomonitoring into environmental surveillance programs will be increasingly crucial for understanding the impact of chronic exposures on human health and adopting timely measures to mitigate risks [19].

HBM has also become a valuable tool for retrospective analyses and post-incident chemical exposure assessments [20], supporting exposure assessments by distinguishing between exposed and non-exposed individuals and estimating exposure levels compared to baseline population data. Moreover, its application has proven particularly valuable in disaster management, where rapid assessment of toxic exposure levels is essential for guiding emergency interventions, medical treatments, and long-term recovery plans [21].

## 2. Environmental Contamination, HBM, and Psychological Factors

Living in an area where studies have revealed the presence of environmental toxicants is a recognized risk factor for both health and psychological well-being [22,23,24,25]. Understanding human exposure to persistent environmental pollutants is critical to informing public health responses and addressing community concerns in contaminated areas. These pollutants, including PFAS, have been associated with several adverse physical and psychological health outcomes throughout the human life stage, including reduced kidney function, metabolic syndrome, thyroid disturbance, cancer, adverse pregnancy outcomes, and chronic distress [17,26,27]. However, beyond the direct health risks, individuals living in PFAS-contaminated areas often experience considerable and prolonged uncertainty regarding the implications for their health and the health of their families [26].

This etiologic uncertainty contributes significantly to psychological distress. Affected individuals may fear developing illnesses in the future due to prolonged exposure to toxicants, and they often struggle to obtain a clear diagnosis for symptoms that appear long after exposure. This lack of diagnostic clarity leads to confusion about appropriate treatment and prognosis, exacerbating anxiety and distress [28]. Moreover, HBM plays a crucial role in this process by providing scientific data on pollutant levels in the human body, helping to evaluate potential health risks and inform policy responses [29]. However, beyond the scientific and regulatory aspects, it is essential to consider how affected communities perceive and experience biomonitoring efforts.

Awareness of HBM results may heighten insecurity and uncertainty [26,27], leading to increased fear about future health outcomes and contributing to psychological distress. The cognitive and emotional burden of interpreting biomonitoring data can further amplify anxiety, especially when individuals are unsure how to contextualize their exposure levels.

The communication of HBM results has been identified as a crucial issue [30,31,32] due to its significant impact on individuals’ psychological responses. When findings indicate exposure to hazardous substances without providing clear guidance on health risks or mitigation strategies, affected individuals may experience heightened worry and distress. Conversely, effective and transparent communication of biomonitoring data can empower individuals [33], allowing them to take proactive steps to reduce exposure, advocate for policy changes, and regain a sense of control over their health. Thus, the way HBM findings are conveyed plays a pivotal role in shaping psychological well-being, influencing both stress levels and the capacity for informed decision-making. Also, the exclusion from HBM led to a profound sense of “health ostracism”, i.e., the perception of being ignored, or left unprotected by institutions concerning one’s health needs [34]. This exclusion triggered a range of negative emotions, including loneliness, abandonment, and concerns about personal well-being. Moreover, it fostered frustration and anger, contributing to deepening distrust toward institutions, as affected individuals perceived a lack of transparency, equity, and responsiveness in public health initiatives and environmental policies. These dynamics have been extensively illustrated in previous in-depth qualitative research conducted in the Veneto Region [34,35].

Along a similar line, Morello-Frosch and colleagues [31] highlighted the ethical and scientific challenges of communicating biomonitoring results. As this technique becomes more widespread, a key question emerges: whether and how to report individual results to study participants, particularly when the health implications remain uncertain. The authors argued that the lack of clear regulatory benchmarks for many chemicals complicates the interpretation of exposure data, raising concerns about how individuals might react to uncertain or ambiguous findings [31]. Similar concerns have been echoed in previous research, which points to the potential psychological distress and anxiety caused by reporting exposure levels without a clear understanding of their health consequences [36]. In response to these challenges, Morello-Frosch and colleagues [31] outlined three main approaches to result communication. Clinical ethics emphasizes reporting results only when the relationship between exposure and health effects is well established. This approach, which has been historically implemented in occupational health studies [37], aims to prevent unnecessary alarm by limiting disclosure to cases where clinical significance is clear. However, this method has been criticized for potentially restricting individuals’ right to know their exposure levels, even in the absence of definitive health risks [38]. In contrast, community-based participatory research (CBPR) takes a more inclusive approach by encouraging affected populations to play an active role in interpreting and responding to exposure data. CBPR fosters engagement by ensuring that study participants receive meaningful information about potential risks while also addressing scientific uncertainties [36]. Morello-Frosch and colleagues [31] noted that this framework requires careful balancing between the community’s right to know and the ethical obligation to prevent unnecessary distress. The third approach, citizen science and ‘data judo’, is often employed by environmental advocacy groups seeking to leverage biomonitoring results for policy change and public awareness. This strategy treats data as a tool for activism, mobilizing affected populations and pressuring policymakers to adopt stricter regulations on hazardous substances. Overall, the debate on biomonitoring result communication underscores the need for clearer ethical guidelines and a participatory approach that balances scientific rigor with the public’s right to information [31].

Additionally, as emerged from a psychosocial study on communities exposed to chronic PFAS contamination [26,34], undergoing biomonitoring is not merely an informative process in which individuals learn about the presence and concentration of environmental chemicals in their bodies. Rather, HBM represents a profound psychosocial transition that reshapes an individual’s relationship with themselves, others, and the identified contaminant, often perceived as an unwelcome and threatening presence, making it a deeply psychological and social experience rather than just a biomedical one.

This psychological transition [26] is often accompanied by a complex emotional journey, including anxiety, fear, frustration, or resignation, as individuals grapple with the uncertainty surrounding their exposure and its potential health consequences.

Finally, public perception plays a crucial role in shaping responses to environmental contamination and biomonitoring initiatives. It can significantly influence trust in health authorities, willingness to participate in studies, and the overall effectiveness of intervention measures [39]. When communities perceive that their concerns are acknowledged and addressed with transparency, trust in institutions is strengthened, which in turn fosters greater cooperation in public health initiatives. Conversely, if communication is perceived as inadequate, dismissive, or contradictory, skepticism and resistance may arise, ultimately undermining health interventions. Therefore, ensuring that affected communities receive clear, accurate, and empathetic communication is essential not only for psychological well-being but also for the successful implementation of public health policies.

### The Present Study: Aims and Hypotheses

The present study focused on the Veneto Region in Italy, where a severe PFAS contamination has been documented [14], affecting local water supplies and raising concerns among residents about potential health consequences [34]. This issue is particularly serious within the so-called ‘Red Area’, a 595 km^2^ zone affected by long-term water and soil contamination from PFAS, primarily attributed to emissions from an industrial plant that has been producing PFAS since 1968, according to official estimates. In 2016, residents of this territory were invited to undergo HBM assessments as part of a health surveillance plan to detect PFAS levels in their blood. Health consequences in the Red Area have been concerning, with reports of physical conditions such as preeclampsia, gestational diabetes, low birth weight in children, cardiovascular disease, and cancer mortality, all of which have been linked to prolonged PFAS exposure [14,40].

In this research, we aimed to explore the health concerns, life satisfaction, and perceptions of individuals living in this territory about the quality of HBM efforts, figuring out how these factors interact and influence each other. Considering the abovementioned literature on the topic and the key role of communicative and relational aspects of HBM screening, we formulated the following research question: how does perceived quality of the HBM process influence the health concerns related to PFAS contamination and the perceived quality of life of individuals living in the most contaminated areas of the Veneto Region? Based on this question, the study hypothesized that:

**H1.1.**  *PFAS Health Concern is negatively correlated to Quality of Life.*

**H1.2.**  *PFAS Health Concern is negatively correlated to HBM Process Satisfaction.*

**H1.3.**  *HBM Process Satisfaction is positively correlated to Quality of Life.*

**H2.1.**  *There are gender differences in PFAS Health Concern, HBM Process Satisfaction, and Quality of Life scores.*

**H2.2.**  *There are differences in PFAS Health Concern, HBM Process Satisfaction, and Quality of Life between subjects with and without children.*

**H3.**  *HBM process satisfaction moderates the relationship between PFAS Health Concern and Quality of Life. Specifically, higher perceived quality of the HBM process is expected to mitigate the negative impact of PFAS health concerns on Quality of Life.*

## 3. Materials and Methods

### 3.1. Study Area: The Red Area

**Figure 1:**
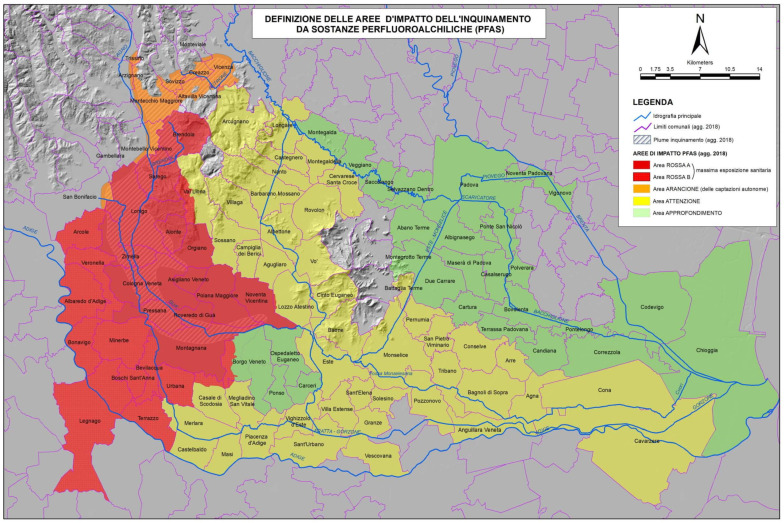
PFAS exposure map showing the different colored areas [43].

Investigations conducted from 2013 to 2016 revealed widespread contamination in both water and soil, affecting communities and ecosystems across three provinces of the Veneto Region: Vicenza, Verona, and Padova. In December 2016, following the reconstruction of the drinking water supply by the local service providers, the Veneto Region categorized the areas affected by the PFAS contamination into four zones based on a risk gradient [41,42], as shown in Figure 1. The Red Area, the most heavily impacted, includes municipalities where PFAS contamination affects public drinking water, well water, surface water, and groundwater, resulting in high exposure for residents. The Orange Area includes municipalities where PFAS contamination was found only in private wells, surface water, and groundwater, with no contamination in public drinking water, leading to moderate exposure. The Yellow Area is under special surveillance, with a monitoring system in place for surface and groundwater, including irrigation and drinking water. Finally, the Green Area contains PFAS solely in environmental matrices, warranting further monitoring and research. We decided to concentrate our analysis on the Red Area, the most affected by PFAS contamination, which currently includes 30 municipalities in an area of about 595 km^2^, and is further subdivided into Red Area A, where contamination is more severe, and Red Area B [14].

### 3.2. Procedure

We administered an exploratory cross-sectional study [44]. The reference population was the adult residents of Red Areas A and B who had undergone biomonitoring. The inclusion criteria for participation in the research were: being over 18 years old, having joined the Surveillance Program for populations exposed to PFAS, and having undergone at least the first level of screening. Data collection occurred from January 2023 to February 2024.

The questionnaire was delivered via the Google Forms platform or in paper format; participants were recruited through a convenience snowball sampling: initially, the questionnaire was shared with the contribution of citizen science from the Mamme NO PFAS group involved in the PFAS issue, who was then encouraged to further invite other people from their network to participate in the study.

### 3.3. Measures

The questionnaire was divided into four sections. Items from previous studies in the international literature were translated and modified for the Italian context with the assistance of a native English speaker. To ensure the questionnaire’s relevance to the research context, a translation and back-translation procedure was carried out when needed, followed by an expert review from specialists in environmental and social psychology. Reliability analyses were performed to assess internal consistency, confirming the robustness of the adapted scales.

#### 3.3.1. Section 1: General Data

Items in this section investigated the socio-demographic data of participants, including gender (woman, man, prefer not to answer), age, marital status (single, married, cohabiting, separated, divorced, widowed), number of children, educational qualification (elementary school diploma, middle school diploma, upper secondary school diploma, bachelor’s degree, master’s degree, PhD), occupation, and area of residence (Red Area A, Red Area B). Health-related items were also included to collect data about the health conditions (no health issues, mild or short-term health issues, significant health issues), the period during which they adhered to HBM, the HBM screening phase (screening, follow-up), professional assistance sought (general practitioner, PFAS expert, counselor or psychologist, psychotherapist), and motivations to undergo HBM (personal health concerns, children and family well-being, information, prevention, contribute to science).

#### 3.3.2. Section 2: PFAS Health Concern

This section included three dichotomous items developed by the research team, based on previous qualitative studies [26,27], to assess the level of health concern regarding PFAS contamination. These were subsequently merged into a composite score (range: 3–6). Participants were asked to answer this question: “Are you concerned about your health or the health of others due to living in an area contaminated by PFAS?” choosing between “yes” (coded with 2) or “no” (coded with 1), depending on whether they were worried about their own, their partner’s and their children’s health or not.

#### 3.3.3. Section 3: HBM Process Satisfaction

This section comprised eight items scored on a 4-point Likert scale, adapted from the General Satisfaction Questionnaire 8 (GSQ-8) [45], a self-report instrument designed to investigate user satisfaction regarding the care received in healthcare settings. It rates the effectiveness of communication and help provided, the acceptability of services, and overall satisfaction. Examples are as follows: “What impression did you have of the welcome you received when you first met the staff?”, “Did you receive the type of service you wanted?”, “Are you satisfied with how the healthcare staff assisted you?”. A PCA was performed on the items’ correlation matrix, which returned only one component, explaining 54.2% of the total variance. The internal consistency of the scale was good (α = 0.88).

#### 3.3.4. Section 4: Quality of Life

This section included the World Health Organization Quality of Life (WHOQOL-BREF) [46], including 26 items on a 5-point Likert scale, designed to assess how the participants perceive their quality of life. Quality of life is a multidimensional construct composed by four domains: (1) physical health, related to the participant’s physical health status (activities of daily living; dependence on medication and medical devices; energy and fatigue; mobility; pain and discomfort; sleep and rest; work capacity); (2) psychological, related to the participant’s psychological condition (perception of physical appearance; negative feelings; positive feelings; self-esteem; spirituality, religion, and personal beliefs; thinking, learning, memory, and concentration); (3) social relationships, related to the participant’s social relationships (personal relationships; social support; sexual activity); (4) environment, related to the economic, social, and environmental context (financial resources; freedom, physical integrity, and safety; quality and accessibility of healthcare and social services; home environment; opportunities to acquire new knowledge and skills; participation in recreational activities; physical environment—pollution, noise, traffic, climate; transport). A PCA was carried out and sustained the preference for a one-component solution (30% of the total variance explained) (α = 0.89).

### 3.4. Data Analysis

When deemed appropriate (e.g., newly devised measures), a Principal Component Analysis (PCA) was performed to support the expected unidimensional factorial structures (Section 3.3.3 and Section 3.3.4 of the questionnaire). Then, we computed Cronbach’s alpha to estimate reliability. Subsequently, we calculated composite scores for the key variables PFAS Health Concern, HBM Process Satisfaction, and Quality of Life, and then we computed the following statistical analyses through IBM SPSS Statistics (v. 29.0.1.0): descriptive statistics on the General Data (Section 3.3.1) and scales (Section 3.3.2, Section 3.3.3 and Section 3.3.4); Pearson’s correlation coefficients between composite scores; MANOVA and *t*-test for checking mean differences between subgroups, and Moderation analysis to test H1.

## 4. Results

### 4.1. Sample Description

The final sample was composed of 84 subjects. Of these, 44 (52.38%) were women and 40 (47.62%) were men; participants were aged 18–65 years (Mean = 43.12, SD = 12.89). Concerning the level of education, seven subjects (8.33%) obtained an elementary or middle school diploma, 51 (60.71%) completed upper secondary school, 12 (14.29%) had a bachelor’s degree, and 13 (15.48%) had a master’s degree.

Regarding marital status, 51 respondents (60.71%) were married, 21 (25.00%) were single, seven (8.33%) were cohabitants, and four (4.76%) were separate. Fifty-six participants (66.67%) had from one to four children (Mean = 1.36, SD = 1.06); 28 (33.33%) participants had no children.

Sixty-eight participants (80.95%) had a working activity. Eight (9.52%) were students, three (3.57%) were housewives, three (3.57%) were retired, one participant (1.19%) was unemployed, and one (1.19%) did not answer.

A total of 69 participants (82.14%) resided in Area Red A, 15 (17.86%) in Area Red B. The years of residence or domicile in the contaminated municipalities ranged from two to 65 years (Mean = 31.79, SD = 16.31). Eighty participants (95.24%) declared themselves to live with others, while four (4.76%) lived alone.

Regarding health status, 38 subjects (45.24%) reported no health issues, 35 participants (41.67%) referred to mild or short-term health problems, while 11 (13.09%) declared more significant health issues.

A total of 28 participants (33.33%) reported seeking professional help to manage their physical or psychological health about PFAS contamination: 24 of them consulted their general practitioner, 14 consulted PFAS experts, two asked a counselor or psychologist, and two a psychotherapist.

Participants underwent biomonitoring screening in the years following the start of the program. Fifty-five respondents (59.52%) reported joining the biomonitoring program due to personal health concerns, 50 (59.52%) for the well-being of their children and family, 49 (58.33%) for more information, 47 (55.95%) for prevention, and 42 (50%) to contribute to science. Sixty subjects (71.43%) joined the first level of biomonitoring (screening), while 24 (28.57%) participated in the second level (follow-up at internal medicine and cardiology clinics, along with additional specialized assessments).

With three predictors, α = 0.05, and a medium effect size (*f*^2^ = 0.15), the application of multiple regression requires a sample of 76 respondents to reach the power of 0.80 [47].

### 4.2. Descriptive Statistics, Reliability Estimates, and Pearson’s Correlation

Table 1 presents descriptive statistics and pairwise correlations for the three examined variables: PFAS Health Concern, HBM Process Satisfaction, and Quality of Life.

Regarding PFAS Health Concern, the mean value (Mean = 5.56) suggests a high level of concern for health risks related to PFAS contamination among participants. The standard deviation (SD = 0.83) and the kurtosis value (K = 2.03) indicate moderate variability in the responses, suggesting that participants’ concerns vary somewhat but are centered around the higher end of the scale. The negative skewness value (SK = −1.77) suggests that most participants reported a high level of concern, with fewer participants expressing low concern.

The HBM Process Satisfaction distribution shows, on average, a moderate level of satisfaction with HBM (Mean = 2.53), with a relatively low variability (SD = 0.58). The data is fairly symmetrical (SK = 0.11), with no clear skew in responses. The kurtosis (K = −0.11) indicates a slightly flatter than normal distribution, suggesting a moderate degree of variation in satisfaction levels but no extreme values.

A generally positive assessment of Quality of Life (Mean = 3.48) emerges, yet not overwhelmingly high. Low variability in responses (SD = 0.46) and a slightly peaked distribution (K = 1.03) indicate that most participants rated their quality of life similarly around the mean. The skewness value (SK = −0.63) suggests that most participants rated their quality of life above the middle of the scale.

Moreover, correlation analysis revealed significant, albeit not high, relationships between the examined variables. In particular, the weak negative relationship between PFAS Health Concern and Quality of Life scores (*r* = −0.25, *p* < 0.05) indicates that participants who express greater health concern about PFAS contamination tend to report slightly low quality of life ratings (H1.1).

The weak negative correlation between PFAS Health Concern and HBM Process Satisfaction (*r* = −0.27, *p* < 0.05) suggests that participants who express more health concern about PFAS contamination tend to report slightly lower satisfaction with the HBM experience (H1.2).

Finally, the small-to-moderate positive correlation between HBM Process Satisfaction and Quality of Life (*r* = 0.30, *p* < 0.01) indicates a mild, positive relationship between perceived quality of the HBM process and overall perceived quality of life (H1.3). Participants who report higher satisfaction with the HBM process also tend to evaluate their quality of life as high. Even if quality of life is a multifaceted and complex construct, this could suggest that having a positive process experience may contribute to a greater sense of life satisfaction.

### 4.3. Differences Between Subgroups

A MANOVA was conducted to check gender differences (Males, *n* = 40; Females, *n* = 44) concerning the mean scores of PFAS Health Concern, HBM Process Satisfaction, and Quality of Life (H2.1). The multivariate effect was significant, *F* (3,80) = 4.88, *p* < 0.01, *η*^2^_p_ = 0.16. The analysis of univariate effects revealed that females declared to be more concerned about PFAS-related health risks (Mean = 5.75 vs. Mean = 5.35; *F* (1,84) = 5.16, *p* < 0.03, *η^2^*_p_ = 0.06), while men were more satisfied for both the HBM Process (Mean = 2.71 vs. Mean = 2.36; *F* (1,84) = 8.10, *p* < 0.01, *η^2^*_p_ = 0.09), and their Quality of Life (Mean = 3.62 vs. Mean = 3.35; *F* (1,84) = 7.98, *p* < 0.01, *η^2^*_p_ = 0.09).

Albeit the overall level of PFAS Health Concern was high, participants with children (*n* = 57) scored higher compared with those without children (*n* = 27), Mean = 5.07 vs. Mean = 5.79, *t* (82) = −4.03, *p* < 0.001, Cohen’s *d* = 0.76 (H2.2).

### 4.4. Moderation Analysis

To test H3, a median split was applied to the supposed moderator variable (M), i.e., “HBM Process Satisfaction”, obtaining two groups: low (*n* = 39, Mean = 2.03, SD = 0.31), coded with 0, vs. high (*n* = 33, Mean = 3.11, SD = 0.32) satisfaction, coded with 1. We opted for a median split approach based on the limited sample size available, the sufficiently symmetrical moderator variable’s distribution, and to simplify both interpretation and presentation of results [48]. A linear regression analysis was run with three predictors—“PFAS Health Concern” (IV), “HBM Process Satisfaction” (M), and their interaction (IVxM)—using SPSS 29. The dependent variable (DV) was “Quality of Life”. The significance of regression coefficients was estimated using a 95% confidence interval (95% C.I.) and the bootstrap procedure with 5000 resamples. The model explained 17.4% of the variance of DV, *F*(3,71) = 4.773, *p* < 0.005. The results are summarized in Table 2.

**Figure 2:**
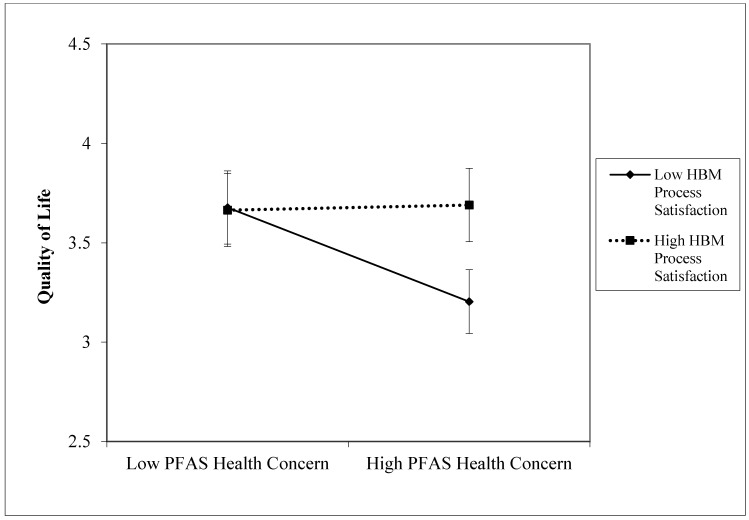
Simple slope analysis. DV = Quality of Life.

Since the interaction turned out to be significant, we proceeded with a simple slope analysis. For the link between “PFAS Health Concern” and “Quality of Life” in case of low “HBM Process Satisfaction”, *b* = −0.271, *t* (68) = −2.28, *p* < 0.03; for high satisfaction, *b* = 0.015, *t* (68) = 0.216, *ns*. Figure 2 depicts this evidence.

In sum, the higher the “PFAS Health Concern” (IV) scores, the lower the “Quality of Life” (DV) scores (Table 1). However, this negative effect significantly worsens when “HBM Process Satisfaction” is low (IV×M). Interestingly enough, high satisfaction with the HBM process seems to be able to attenuate the negative effect of high health-related concern for PFAS contamination on quality of life.

## 5. Discussion

In this exploratory cross-sectional study, we aimed to investigate how health concerns related to PFAS contamination and satisfaction with the biomonitoring process influenced the perceived quality of life in the context of serious environmental contamination. To this end, we administered a questionnaire among residents of the Red Area—the most seriously affected by the PFAS contamination in the Veneto Region—who have adhered to the public HBM program.

The results of this study provided valuable yet preliminary insights into how PFAS Health Concern, HBM Process Satisfaction, and Quality of Life may interact within communities affected by chronic environmental contamination in the Veneto Region.

First, we found that most participants were very concerned about the health risks associated with PFAS contamination. This datum reflects the awareness and apprehension in the affected population, especially under a condition of chronic uncertainty regarding personal and family health implications [26]. Like other studies on environmental stressors have shown, chronic exposure to toxic substances, in addition to their well-documented risks of organic pathologies [14,17,40], can contribute to increased anxiety, uncertainty, and reduced well-being [17,23,26,28]. Higher PFAS Health Concern scores were registered among females compared to males. This finding is in line with previous research showing higher distress due to the consequences of environmental violence in women compared to men, e.g., [49], and qualitative studies discussing gender-based activism [27] in the context of the PFAS environmental contamination in Veneto. We also found that participants with children were, on average, more worried than participants without children. This is consistent with the literature, which shows that parents tend to be more exposed to the psychosocial consequences of contamination, since they have to deal with concerns about their children’s health and future [26,50]. It cannot be ruled out that all this may have serious negative consequences on perceived self-efficacy and self-esteem over time [51]. Moreover, it may generate a pernicious sense of guilt in parents, since they may feel unable to protect their children from PFAS contamination. Future studies could be designed to verify these potential short- and long-term detrimental issues and hypothesize appropriate psycho-socio-educational intervention paths.

We found a weak negative correlation between health concerns about PFAS contamination and perceived quality of life, such that higher levels of health concern about PFAS contamination were associated with lower quality of life ratings. These emotional responses likely reflect the distress linked with both the direct health risks and the uncertainty about long-term health outcomes [26,34].

However, we point out that participants tended to report a relatively positive perceived quality of life, with males reporting higher satisfaction. Notably, this variable is a multidimensional construct encompassing physical, psychological, socio-economic, and environmental facets of an individual’s life. Each of these dimensions contributes to an individual’s overall perceived quality of life, and they can influence how people rate their well-being in different contexts. Despite potential concerns about health risks related to PFAS, other aspects, such as emotional resilience, social support, and access to resources, may be associated with a more positive perception of quality of life and potentially mitigate the emotional burden of these worries. Future prospective or intervention-based research studies could explore whether environmental contamination, such as exposure to PFAS, influences perceived quality of life across different socio-economic groups or social capital levels. Specifically, research could investigate how concerns about the negative health effects of PFAS may differentially impact the various dimensions of quality of life—physical, psychological, socio-economic, and environmental—unpacking potential variations in the way these concerns affect individuals based on their resources or social support networks.

The participants’ overall evaluation of the HBM process was moderate (with a significant difference in favor of males), indicating a generally neutral to slightly positive assessment of their experience with the process. The observed gender differences may be partially influenced by gendered perceptions and expectations of care quality. Women and men may perceive or judge the same healthcare experience differently, based on socially shaped expectations about care, communication, and empathy. Future studies should further explore how gendered expectations shape perceptions of institutional responses in environmental health settings.

We found a weak negative correlation between PFAS Health Concern and HBM Process Satisfaction. That is to say that participants who reported higher levels of concern about PFAS contamination tended to express slightly lower satisfaction with the HBM process. This could suggest that individuals with more significant health concerns related to PFAS may have higher expectations or more anxiety surrounding the HBM process, and thus, may be seeking more detailed information, reassurance, or clarity about the potential health risks associated with their exposure. If these needs are not fully addressed by healthcare professionals, it could result in lower satisfaction and exacerbated anxiety. Knowledge of HBM results can amplify feelings of insecurity and uncertainty [26,34], heightening fears about potential future health issues and psychological distress, especially when individuals are unsure how to contextualize their exposure levels and no indications on health risks or mitigation strategies are provided [33]. This highlights the potential benefits of tailored care approaches and risk communication strategies to meet the expectations and needs of different user groups [32,36], actively involving them in healthcare planning [52]. However, since the correlation found is weak and the study sample was small, this relation should be interpreted with caution. Future research with larger, more diverse samples is needed to better understand this implication.

We also found a small-to-moderate positive correlation between satisfaction with the HBM process and quality of life. Participants who were more satisfied with their experience of biomonitoring tended to report a higher quality of life.

Moreover, our third hypothesis, that HBM Process Satisfaction moderates the relationship between PFAS Health Concern and Quality of Life, was confirmed. Specifically, we found that higher satisfaction with the process attenuated the negative effects of PFAS health concerns on quality of life. This interaction highlighted a potential buffering effect of the quality of care received in terms of perceived personnel’s competence, empathetic support, and communication skills in mitigating the distress provoked by environmental contamination, reinforcing previous evidence about the critical role of communication and relational modes throughout the whole HBM process [31,52]. Yet, this result requires robust confirmation through further research encompassing a large and stratified sample.

The contribution of HBM to prevention and public health protection has been recognized at the European level [18], alongside the need to balance the community’s right to know with the ethical responsibility to prevent unnecessary distress, especially when effective mitigation strategies are limited [31]. Careful communication is essential not only to inform individuals about their exposure but also to empower them to make decisions that protect their health and advocate for systemic change [33]. This includes clearly explaining the limits of scientific knowledge, the uncertainties surrounding long-term risks, and the available protective actions. Moreover, HBM has been acknowledged as a significant biographical turning point for affected communities [26]. As such, our results stress the increasing importance for policymakers and healthcare managers to carefully focus on the communication and relational aspects of public surveillance programs. Hence, it is crucial to provide healthcare personnel with comprehensive training that equips them with the necessary interpersonal skills and a deep understanding of the health implications of PFAS contamination. This will enable them to effectively support users by offering clear, accurate information, addressing concerns, and recommending preventive behaviors to limit exposure and reduce risk [31,52,53]. Moreover, developing guidelines for ethically grounded communication, drawing on principles from risk communication, trauma-informed care, and environmental justice, is crucial for public health institutions addressing environmental contamination. Finally, following the tradition of the CBPR, the active involvement of affected citizens in the process of co-producing healthcare services may be a promising way to craft citizen-centric and context-sensitive HBM programs, at the same time, empowering citizens to regain a sense of control over their health [54,55,56,57].

## 6. Limitations

Our study displays several limitations that must be acknowledged. First, the small sample size (*n* = 84) is a significant drawback that impacts the generalizability and robustness of our findings within the broader reference population. Small samples are more prone to sampling error and less likely to detect smaller effects, which can compromise the reliability of the results. This limitation may be explained by the sensitive nature of the health issues related to PFAS contamination, compounded by political implications and ongoing legal proceedings, which could have discouraged potential participants despite the assurance of anonymity. To enhance the validity and generalizability of the findings, future studies should include larger, more diverse samples that can better reflect the variety of perspectives and experiences within these communities.

Second, we used a convenience snowball sampling method, which, in our case, may have led to the overrepresentation of more engaged individuals who had stronger opinions and were concerned about the issue of PFAS contamination (e.g., activism bias), potentially polarizing the findings. Future research could benefit from employing random or stratified sampling methods to ensure a more representative sample that accurately reflects the broader community’s experiences and attitudes.

Third, parental status was assessed as a binary variable (having children vs. not), which may not fully capture the complexity of caregiving responsibilities or diverse family configurations. We acknowledge that this operationalization may oversimplify more nuanced caregiving roles and family structures; hence, future studies should consider more inclusive measures of family roles and dependents.

Although the WHOQOL-BREF is a validated multidimensional instrument for assessing quality of life, given the small sample size, in this study, we used only the overall score to ensure interpretability. Future research with larger and more statistically powered samples should consider disaggregating the WHOQOL-BREF to explore potentially meaningful differences across specific domains (e.g., physical, psychological, social, and environmental).

Moreover, this study was correlational, meaning we could not confirm the direction of the relations found. The observed correlations may reflect underlying factors that were not captured in the study, such as participants’ socio-economic status, prior health conditions, or levels of community support. Additionally, the relationships could be bidirectional or influenced by other unmeasured variables. Future research should employ longitudinal studies or experimental designs that can track changes over time or manipulate variables to observe causal effects.

Furthermore, all variables in this study were measured through self-reported items and scales, which introduces the potential for response biases. For instance, participants may have over- or underreported their health concerns or overreported their satisfaction with the HBM process due to social desirability bias or a desire to align with perceived expectations.

Another limitation concerns the measurement of PFAS Health Concern, which was assessed using three dichotomous items (cf. Section 3.3.2). Given the multidimensional nature of perceived health risk, including cognitive, emotional, and social components, future research would benefit from adopting validated multi-item scales to improve research depth and sensitivity.

Finally, to reduce respondent burden, we included only a limited set of variables previously linked to the psychological impact of environmental contamination. Consequently, the moderation model explained a modest 17.4% of the variance in quality of life, suggesting that other relevant factors were not captured. Future research should consider broader psychosocial and contextual variables, such as coping styles, institutional trust, and social support, to better reflect the complexity of responses to chronic exposure.

## 7. Conclusions

This exploratory cross-sectional study provides an initial understanding of how satisfaction with the HBM process could influence the perceived quality of life in the context of chronic environmental contamination by offsetting the negative effect of health concerns related to PFAS.

These results contribute to the growing body of literature on the psychological and social impacts of environmental contamination. They emphasize the need for a holistic approach in public health interventions that consider both the physical and emotional dimensions of exposure. The positive role of HBM Process Satisfaction in enhancing Quality of Life suggests that future health interventions should focus on improving the quality of interactions between health professionals and affected communities, as this can significantly mitigate the emotional burden associated with environmental risks. For example, integrating psychological support into environmental health programs may be an effective strategy for reducing the adverse effects of contamination-related anxiety. Clear, transparent, and contextualized communication about biomonitoring results, a helpful and empathetic attitude, and thorough information about risk reduction strategies have been proven to be essential for minimizing psychological distress and fostering trust in health authorities [31,52,53]. Particular attention should be given to providing tailored support to parents, especially mothers, who often carry a heightened emotional and caregiving burden in the context of environmental health crises. Consequently, it may not be useless to keep children’s anxiety levels monitored in the long term, using validated instruments, e.g., [58]. Moreover, community leaders and policymakers should consider adopting community-based participatory approaches (CBPR) to improve engagement with affected populations and ensure that public health responses are inclusive and responsive to community concerns. Addressing community perceptions is essential for fostering trust, improving engagement, and ensuring that public health responses are both transparent and equitable [54,59].

Studies with wider and stratified samples or with public registry-based recruitment are expected to enhance the validity of the identified relations. Future projects may include longitudinal designs to offer a more nuanced understanding of how health concerns evolve and are mitigated by ongoing health interventions. Furthermore, studies examining the role of socio-demographic factors, such as gender, education, and socioeconomic status, in moderating the relationship between environmental concern and quality of life would be valuable in designing more targeted and equitable public health strategies. Finally, qualitative research adopting in-depth interviews, focus groups, and participatory methods such as community-based and citizen science-like initiatives could help detail the specific local perceptions, needs, and expectations related to the HMB program, at the same time empowering the affected communities.

## Figures and Tables

**Table 1:** Descriptive statistics and Pearson’s correlation among variables.

Variables	Min	Max	M (SD)	SK	K	2	3
1 PFAS Health Concern	3.00	6.00	5.56 (0.83)	−1.77	2.03	−0.27 * [−0.46; −0.06]	−0.25 * [−0.46; −0.03]
2 HBM Process Satisfaction	1.25	4.00	2.53 (0.85)	0.11	−0.11	-	0.30 ** [0.09; 0.49]
3 Quality of Life	2.15	4.62	3.48 (0.46)	−0.63	1.03		-

Note: * *p* < 0.05; ** *p* < 0.01. Min—minimum; Max—maximum; M—mean; SD—standard deviation; SK—skewness; K—kurtosis; 95% CIs in square brackets.

**Table 2:** Moderation analysis (*n* = 84). DV = Quality of Life.

Variables	*b* (se)	95% CI	*t*	*p*<
PFAS Health Concern (IV)	−0.259 (0.112)	[−0.488, −0.051]	−2.313	0.03
HBM Process Satisfaction (M)	−1.336 (0.762)	[−2.847, 0.160]	−1.754	*ns*
IVxM	0.285 (0.134)	[0.021, 0.553]	2.119	0.04
R^2^ = 0.174				

## Data Availability

The dataset is available from the authors upon reasonable request.
